# Differences in gene expression and cytokine production by crystalline vs. amorphous silica in human lung epithelial cells

**DOI:** 10.1186/1743-8977-9-6

**Published:** 2012-02-02

**Authors:** Timothy N Perkins, Arti Shukla, Paul M Peeters, Jeremy L Steinbacher, Christopher C Landry, Sherrill A Lathrop, Chad Steele, Niki L Reynaert, Emiel FM Wouters, Brooke T Mossman

**Affiliations:** 1 Department of Pathology, University of Vermont College of Medicine, 89 Beaumont Avenue, Burlington, VT 05405, USA; 2 Department of Respiratory Medicine, Maastricht University Medical Centre, Maastricht University, Maastricht, The Netherlands; 3 Department of Chemistry, University of Vermont, Burlington, VT 05405, USA; 4 Department of Medicine, University of Alabama at Birmingham School of Medicine, Birmingham, AL 35233, USA

## Abstract

**Background:**

Exposure to respirable crystalline silica particles, as opposed to amorphous silica, is associated with lung inflammation, pulmonary fibrosis (silicosis), and potentially with lung cancer. We used Affymetrix/GeneSifter microarray analysis to determine whether gene expression profiles differed in a human bronchial epithelial cell line (BEAS 2B) exposed to cristobalite vs. amorphous silica particles at non-toxic and equal surface areas (75 and 150 × 10^6^μm^2^/cm^2^). Bio-Plex analysis was also used to determine profiles of secreted cytokines and chemokines in response to both particles. Finally, primary human bronchial epithelial cells (NHBE) were used to comparatively assess silica particle-induced alterations in gene expression.

**Results:**

Microarray analysis at 24 hours in BEAS 2B revealed 333 and 631 significant alterations in gene expression induced by cristobalite at low (75) and high (150 × 10^6^μm^2^/cm^2^) amounts, respectively (p < 0.05/cut off ≥ 2.0-fold change). Exposure to amorphous silica micro-particles at high amounts (150 × 10^6^μm^2^/cm^2^) induced 108 significant gene changes. Bio-Plex analysis of 27 human cytokines and chemokines revealed 9 secreted mediators (p < 0.05) induced by crystalline silica, but none were induced by amorphous silica. QRT-PCR revealed that cristobalite selectively up-regulated stress-related genes and cytokines (*FOS, ATF3, IL6 *and *IL8*) early and over time (2, 4, 8, and 24 h). Patterns of gene expression in NHBE cells were similar overall to BEAS 2B cells. At 75 × 10^6^μm^2^/cm^2^, there were 339 significant alterations in gene expression induced by cristobalite and 42 by amorphous silica. Comparison of genes in response to cristobalite (75 × 10^6^μm^2^/cm^2^) revealed 60 common, significant gene alterations in NHBE and BEAS 2B cells.

**Conclusions:**

Cristobalite silica, as compared to synthetic amorphous silica particles at equal surface area concentrations, had comparable effects on the viability of human bronchial epithelial cells. However, effects on gene expression, as well as secretion of cytokines and chemokines, drastically differed, as the crystalline silica induced more intense responses. Our studies indicate that toxicological testing of particulates by surveying viability and/or metabolic activity is insufficient to predict their pathogenicity. Moreover, they show that acute responses of the lung epithelium, including up-regulation of genes linked to inflammation, oxidative stress, and proliferation, as well as secretion of inflammatory and proliferative mediators, can be indicative of pathologic potential using either immortalized lines (BEAS 2B) or primary cells (NHBE). Assessment of the degree and magnitude of these responses *in vitro *are suggested as predictive in determining the pathogenicity of potentially harmful particulates.

## Background

Occupational and environmental exposure to fine and ultrafine particulates is rapidly becoming an overwhelming area of concern. With increasing numbers and compositional heterogeneity of potentially harmful natural and synthetic particulates, there is a vital need for screening assays to determine their potential pathogenicity. Crystalline silica particles are known to cause silicosis (a pneumoconiosis) and have other detrimental respiratory effects when inhaled in excessive amounts [1]. Airborne exposures are also associated with lung inflammatory diseases, increased susceptibility to infection, as well as lung cancers, especially in smokers [2,3]. Crystalline silica was stated to be a Class I carcinogen (IARC 1997) which was recently restated (IARC 2010) [4], though epidemiologic data are inconsistent, and its carcinogenic potential in non-smokers remains controversial [5]. Exposure to crystalline silica is associated with industries and occupations including, sandblasting, ceramics, cement manufacture, construction, and quarry and foundry workers [2,6-9]. Although crystalline silica exists in many different polymorphs, those of particular concern are the naturally occurring polymorphs quartz, cristobalite and tridymite [10]. Cristobalite was used in studies here because of its bioreactivity in a rat inhalation model [11].

Inhalation of crystalline silica or other pathogenic minerals such as asbestos and airborne particulate matter (PM) results in lung injury by mechanisms, which are not well understood [12,13]. Crystalline silica particles induce extensive inflammatory and proliferative effects *in vitro *and *in vivo *[14-20]. Activation of several signaling pathways, including the mitogen-activated protein kinases (MAPKs) [21-23] as well as transcription factor complexes including activator protein-1 (AP-1) [24] and nuclear factor kappa B (NFκB), are thought to contribute to the pro-inflammatory, toxic and mitogenic effects of silica [25]. Understanding the broad spectrum of initial events induced by particulate-cell interactions is crucial to revealing the molecular mechanisms that contribute to inflammation, abnormal proliferation and cross-talk between epithelial cells and other cell types in the lung.

Here we focused on lung epithelial cells, which initially interact with respirable particles after inhalation to provide insight into molecular events initiating silica-induced disease processes. Since surface area, as opposed to equal mass concentration, has been shown to be a more appropriate parameter of dosing to determine the effects of fine and ultrafine particulates in biological systems [26-29], human bronchial epithelial cells were exposed to crystalline silica (cristobalite) or amorphous silica (synthetic, dense silica spheres) at non-toxic and equal surface area concentrations. As previous studies from our group have shown that the magnitude and duration of gene expression alterations in human mesothelial cells by asbestos fibers may be predictive of their pathogenic potential [30-32], we utilized robust microarray profiling to compare responses of human lung epithelial cells to pathogenic crystalline vs. amorphous silica. Our findings link the extent of changes in gene expression and cytokine production to silica crystallinity and pathogenicity in silicosis.

## Results and Discussion

### Cristobalite and amorphous silica particles have equal effects on viability of BEAS 2B cells

**Figure 1:**
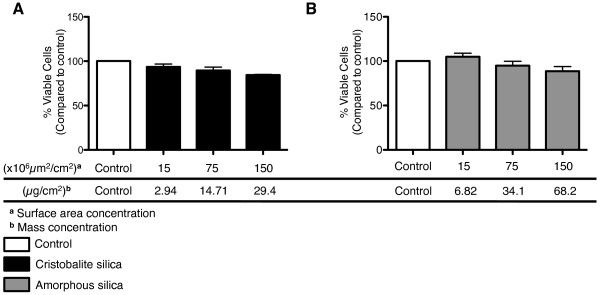
**Assessment of BEAS 2B cell viability after exposure to silica particles**. Cell viability assessed by the Trypan blue exclusion assay of cells exposed to cristobalite (**A**) and amorphous silica particles (**B**) for 24 h. Results are expressed as the mean percent viable cells ± SEM compared to unexposed controls and are representative of 3 independent experiments (N = 3 for each group in each experiment). **^a ^**denotes the surface area concentrations expressed as (x10^6^μm^2^/cm^2^) and **^b ^**denotes the respective mass concentrations of particles used (μg/cm^2^). Concentrations presented are represented by surface area for all other figures and tables.

Surface area is a better metric than mass, thus doses have been measured on a surface area basis as the biological activity of particles is largely based on their respective surface characteristics [29,33-35]. Much of this is accredited to the ability of pathogenic particles to produce reactive oxygen and nitrogen species (ROS and RNS) [36] that promote inflammatory responses and cellular injury [37]. The surface areas of cristobalite and amorphous silica particles (dense silica micro-spheres) were determined by BET nitrogen adsorption analysis [32]. Table 1 presents the surface area/unit mass (m^2^/g) and other properties of cristobalite and amorphous silica particles, including chemical composition (SiO_2_), which is confirmed in (Additional file 1) by SEM-EDS analysis. In addition, the size distribution of cristobalite and amorphous silica particles was determined (Additional file 2). It should be noted that in these studies, a specific type of amorphous silica was used (micron-sized, dense, spheres synthesized by hydrolysis reactions) and therefore conclusions may not necessarily be extrapolated to other amorphous silica types. To avoid use of lethal concentrations of particles, we first performed cell viability assays over a range of concentrations. Both particles had dose-responsive effects on viability, inducing only about 15% loss of viability (non-significant) at highest concentrations (150 × 10^6^μm^2^/cm^2^) over a 24 h period (Figure 1). Both a low (75 × 10^6^μm^2^/cm^2^) and high dose (150 × 10^6^μm^2^/cm^2^) of crystalline silica particles and a high dose (150 × 10^6^μm^2^/cm^2^) of amorphous silica were evaluated by microarray and Bio-Plex analysis for gene expression and cytokine/chemokine secretion, respectively.

**Table 1:** Silica particle characterization

Silica Particle	**Chem. Comp**.	% Purity	S.A. (m^2^/g)^a^	Mean size (μm)^b^	Particles/mg	Source
Cristobalite	SiO_2_	*95.5 ± 0.03	5.1	2.16 ± 2.00	-	C&E Mineral Corp, King of Prussia, PA
Amorphous	SiO_2_	> 99	2.2	3.55 ± 0.45	20.33 × 10^6^	University of Vermont, Dept. of Chemistry

Table 1 summarizes silica particle characterization of cristoablaite and amorphous silica particles.

**^a ^**S.A. = Surface area/mass (m^2^/g).

**^b ^**Mean particle diameter ± standard deviation

* Sample contains traces of quartz (4.54 ± 0.08%)

### Cristobalite and amorphous silica particles interact with and are taken up by BEAS 2B cells

**Figure 2:**
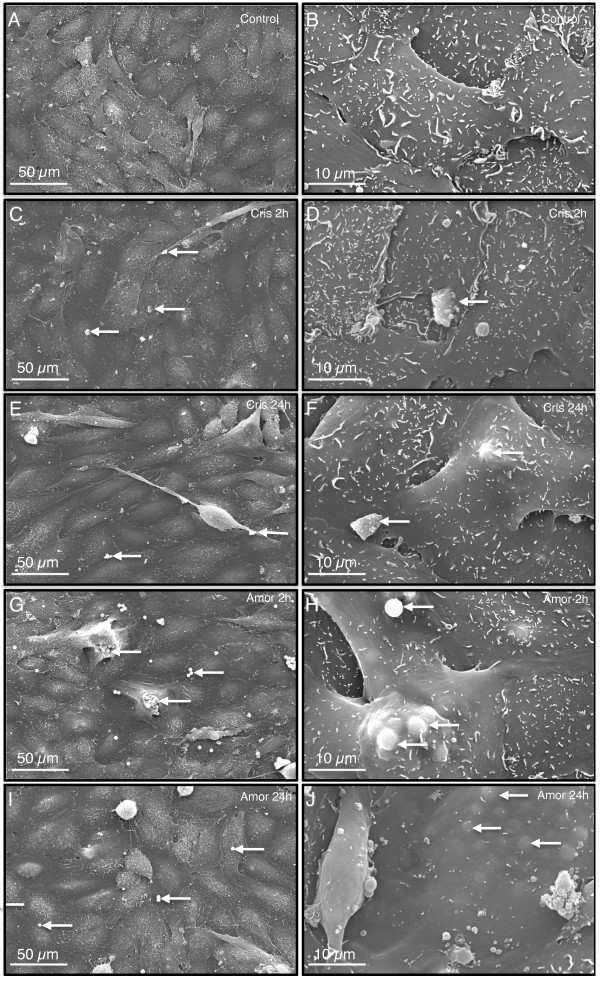
**Interaction and uptake of silica particles by BEAS 2B cells**. Scanning Electron Micrographs of BEAS 2B cells. Unexposed controls (**A, B**), exposed to cristobalite (75 × 10^6^μm^2^/cm^2^) for 2 h (**C, D**), 24 h (**E, F**) and amorphous silica (75 × 10^6^μm^2^/cm^2^) for 2 h (**G, H**) and 24 h (**I, J**). Panels on the left are at low magnification (500×) scale bar = 50 μm and on the right at high magnification (2500×) scale bar = 10 μm. White arrows indicate silica particles.

To determine whether human lung epithelial cells took up both particle types, scanning electron micrographs of BEAS 2B cells exposed to silica particles (75 × 10^6^μm^2^/cm^2^) were examined at 2 and 24 h (Figure 2). The presence of silica particles was confirmed by electron dispersion spectroscopy (EDS) analysis, in which a spectrum was taken of a point "on-particle" showing a strong silicon (Si) peak and an "off-particle" spectrum, which contained no (Si) peak. Both cristobalite and amorphous silica particles interacted with cells at 2 h and were internalized by 24 h (Figure 2). No major toxic effects were observed, correlating with results of cell viability studies. Based on these results, we investigated alterations in gene expression and mediator secretion at these non-toxic particle doses.

### Microarray analysis reveals robust changes in gene expression profiles in BEAS 2B cells exposed to cristobalite vs. amorphous silica particles

**Figure 3:**
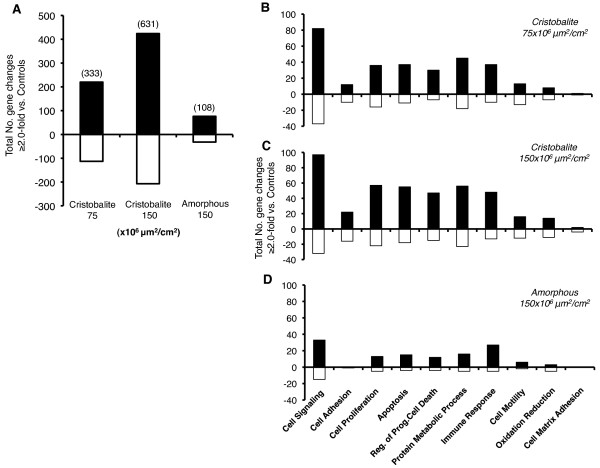
**Microarray analysis of BEAS 2B cells in response to silica particle exposure (24 h)**. (**A**) Total number of significant gene changes (p < 0.05) with a cut-off of ≥ 2.0-fold change in mRNA compared to unexposed controls in BEAS 2B exposed for 24 h (total number of gene changes). Gene ontology analysis of BEAS 2B exposed to (**B**) Cristobalite at 75 × 10^6^μm^2^/cm^2^, (**C**) 150 × 10^6^μm^2^/cm^2 ^and (**D**) amorphous silica at 150 × 10^6^μm^2^/cm^2^. Positive values represent number of genes up-regulated (Black bars) and negative values represent number of genes down-regulated (white bars) (N = 3).

It has been shown both *in vitro *and *in vivo *that crystalline silica particles are more biologically active and pathogenic than amorphous silica particles [20,38-40]. Therefore, we sought to determine if there were significant differences in how different silica particles (crystalline vs. amorphous) would affect gene expression and cytokine/chemokine secretion at equal surface area concentrations in the BEAS 2B immortalized cell line. Affymetrix/GeneSifter analysis of BEAS 2B cells exposed to cristobalite silica at 75 and 150 × 10^6^μm^2^/cm^2 ^were compared to patterns using amorphous silica particles at 150 × 10^6^μm^2^/cm^2 ^for 24 h, the time point of maximum gene expression observed with pathogenic particulates in previous studies using human mesothelial cells exposed to asbestos [30-32], primary human airway epithelial cells exposed to PM fractions [41] or chrysotile asbestos [42]. A dose-responsive pattern was observed with cristobalite silica that induced a total of 333 and 631 significant gene changes at 75 and 150 × 10^6^μm^2^/cm^2^, respectively (p < 0.05 with a cut-off of ≥ 2.0-fold change compared to controls). In addition, expression of certain genes was dose-responsive as well. For instance, matrix metallopeptidase 1 (*MMP1*) gene expression was increased 16-fold and 44-fold at 75 and 150 × 10^6^μm^2^/cm^2^, respectively (Table 2). This dose responsive trend was observed overall in mRNA levels of the 10 most highly expressed genes after cristobalite exposures as presented in Table 2. However, amorphous silica particles, at the highest surface area concentration (150 × 10^6^μm^2^/cm^2^), induced only 108 significant gene changes, nearly 6-fold less than cristobalite silica (Figure 3A). Of the 108 changes induced by amorphous silica, 93 of these changes were also induced by cristobalite silica at the same concentration.

**Table 2:** Top 10 genes affected by 24 h exposure to silica particles in BEAS 2B

Gene Name (Abbreviation)	Fold Change^a^		
	Cristobalite		Amorphous
	75^b^	150^b^	150^b^
**Up-regulated ▲**			
Myxovirus (influenza virus) resistance 2 (mouse) (**MX2**)	20.86	73.76	22.33
Matrix metallopeptidase 1 (interstitial collagenase) (**MMP1**)	16.12	44.68	18.75
Sodium Channel, voltage-gated, type III, beta (**SCN3B**)	15.33	20.54	NC
2'-5'-oligoadenylate-synthetase 2, 69/71kDa (**OAS2**)	13.56	34.77	12.17
Myxovirus (influenza virus) resistance 1, interferon-inducible protein p78 (mouse) (**MX1**)	9.61	23.33	8.72
Interferon-induced protein 44-like (**IFI44L**)	7.64	22.82	8.63
Interferon-induced protein 44 (**IFI44**)	7.51	19.90	8.24
B-cell Linker (**BLNK**)	7.24	9.32	NC
BTG family, member 2 (**BTG2**)	7.22	8.56	NC
Carcinoembryonic antigen-related cell adhesion molecule 1 (**CEACAM1**)	7.19	11.05	NC
2'-5'-oligoadenylate synthetase 1, 40/46kDa (**OAS1**)	7.09	20.53	6.75
Interferon, alpha-inducible protein 27 (**IFI27**)	6.59	17.62	6.15
2'-5'-oligoadenylate synthetase-like (**OASL**)	6.80	18.85	6.92
Radical S-adenosyl methionine domain containing (**RSAD1**)	5.45	23.25	6.09
Interleukin 24 (**IL24**)	3.88	8.54	6.47

**Down-regulated ▼**			
Collagen, type I, alpha 2 (**COL1A2**)	6.08	10.86	2.12
Hypoxia inducible factor 3, alpha subunit (**HIF3A**)	4.51	7.18	3.94
Collagen, type I, alpha 1 (**COL1A1**)	4.17	5.52	NC
Methyltransferase like 7A (**METTL7A**)	4.11	5.67	2.89
Cytochrome P450, family 4, subfamily B, polypeptide 1 (**CYP4B1**)	3.93	5.55	3.07
Olfactomedin-like 3 (**OLFML3**)	3.91	5.28	2.06
Mitogen-activated protein kinase kinase 6 (**MAP2K6**)	3.86	4.78	3.21
Collagen, type XI, alpha 1 (**COL11A1**)	3.85	5.46	NC
Potassium inwardly-rectifying channel, subfamily J, member 16 (**KCNJ16**)	3.78	7.11	3.49
Cannabinoid receptor 1 (brain) (**CNR1**)	3.75	5.44	3.48
Calcium channel, voltage-dependent, alpha 2/delta subunit 3 (**CACNA2D3**)	3.53	3.49	2.65
Solute carrier family 38, member 4 (**SLC38A4**)	3.16	3.76	2.65
Leucine rich repeat containing 17 (**LRRC17**)	3.14	5.07	3.61
Protocadherin 9 (**PCDH9**)	2.98	3.69	2.65

Top 10 genes in BEAS 2B cells up and down-regulated by 24-hour exposure to cristobalite silica at 75 and 150 × 10^6^μm^2^/cm^2 ^and amorphous silica particles at 150 × 10^6 ^μm^2^/cm^2^.

**^a ^**Fold change (mRNA) expression (p < 0.05) cut-off ≥ 2.0-fold compared to controls (n = 3).

**^b ^**Silica particle concentrations: (× 10^6^μm^2^/cm^2^)

NC = No Change

Gene ontology (GO) analysis then was used to categorize changes in gene expression caused by silica particle exposures (Figure 3B-D). Ten categories of interest (cell signaling, cell adhesion, cell proliferation, apoptosis, regulation of programmed cell death, protein metabolic processes, immune response, cell motility, oxidation-reduction, and cell matrix adhesion) were used to classify gene changes [30-32]. Interestingly, patterns of both cristobalite silica and amorphous silica-induced mRNA expression were similar, with most gene expression alterations related to cell signaling and proliferation, apoptosis, protein metabolic processes and immune responses. Though the intensity of gene changes (both in number and magnitude or fold-change) was dose-dependent, overall the same patterns of change were seen (Additional file 3). These five categories account for 77, 73 and 80% of gene changes in BEAS 2B cells exposed to cristobalite at 75 and 150 × 10^6^μm^2^/cm^2 ^and amorphous silica at 150 × 10^6^μm^2^/cm^2^, respectively.

Table 2 lists the top 10 gene changes induced by cristobalite vs. amorphous silica. The majority of the top 10 up-regulated genes are related to immune response (*OAS1, OAS2, OASL, IFI44, IFI44L, BLNK*), apoptosis (*MX1, BTG2, IL24 *and *IFI27*) cell signaling (*MX2*) and dissolution of extracellular matrix (*MMP1*). These genes may contribute to the development of silicosis or, in the case of *MMP1*, repair of lung injury. Similar changes in gene expression were observed in a recent study investigating common gene expression responses of macrophages to a number of pathogens including toll-like receptor (TLR) agonists, *Salmonella*, and silica nanoparticles in which the authors described a "macrophage core response module" [43]. Common changes compared with results in Table 2 include: *MX1, MX2, OAS1, OAS2*, and *IFI44*. In our studies, several other genes included in this "macrophage core response module" were also altered in expression (p < 0.05) by cristobalite including *EGR1, EGR2, FOS, GADD45B, IFIT1, IFIT2, JUN, MAFB, PLAU*, and *PTGS1*. These results indicate that foreign materials consistently affect a number of common genes in both macrophages and lung epithelial cells. The specific roles these genes play in the development of lung disease is not well understood; however, they appear to be elicited in acute responses to foreign materials and infectious agents. How these rapid responses affect epithelial cells and macrophages, respectively as well as cross-talk between these cells, is not understood, but they may be important, initial mechanisms of cell response to crystalline silica.

### Steady state mRNA levels of stress-related and inflammatory genes are induced early and selectively by cristobalite in BEAS 2B cells

Based on the results of our microarray analyses, we investigated some genes of interest using qRT-PCR to confirm changes found in microarrays as well as to determine their time-course of induction in BEAS 2B cells. Genes investigated (up-regulated by cristobalite at 75 and 150 × 10^6^μm^2^/cm^2 ^in microarray) included the early response gene *FOS *(2.12, 3.51-fold), a member of the activator protein-1 (AP-1) transcription factor complex [44], activating transcription factor 3 *ATF3 *(2.68, 3.16-fold), which is up-regulated in gene-profiling by several particulates in human mesothelial cells and primary bronchial epithelial cells [30-32,42], and the pro-inflammatory cytokines *IL6 *(2.12, 2.59-fold) and *IL8 *(2.12, 2.65-fold), both shown to be up-regulated by crystalline silica in A549 cells and a number of *in vitro *and *in vivo *models [21,45-49].

**Figure 4:**
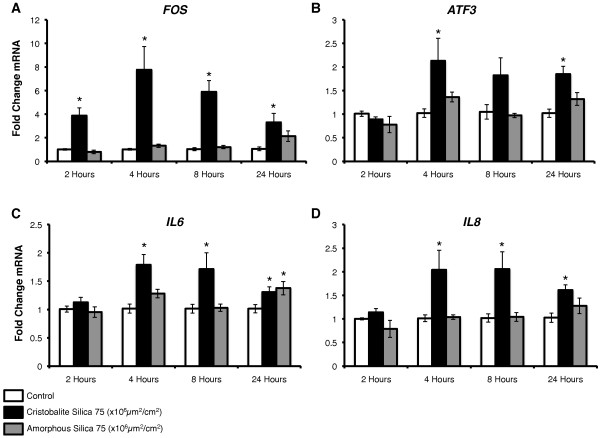
**BEAS 2B steady state mRNA levels over time with exposure to silica particles of stress-related and immune response genes**. Time-course analysis of mRNA levels by QRT-PCR of BEAS 2B cells exposed to 75 × 10^6^μm^2^/cm^2 ^cristobalite (black bars) and amorphous silica (gray bars) and unexposed controls (white bars) for 2, 4, 8 and 24 h. Fold change in mRNA of (**A**) *FOS*, **(B**) *ATF3*, (**C**) *IL6 *and (**D**) *IL8*. *P < 0.05 compared to unexposed controls. Bars denote mean ± SEM. Results from two independent experiments were pooled (N = 6/group/time-point). Values of cells exposed to amorphous silica at 2 and 4 hours (N = 5/group/time-point).

In all cases, mRNA levels of these genes were only up-regulated significantly by crystalline in contrast to amorphous silica (Figure 4). Consistent with its role as an early response gene, *FOS *expression was significantly increased at 2 h and peaked at 4 h post-exposure to silica (Figure 4A). These findings indicate that stress-response genes are rapidly activated by crystalline silica but not by amorphous silica particles in human lung epithelial cells. It has previously been demonstrated that *FOS *expression is increased by alpha quartz (DQ12 and Min-U-Sil), and coal-mine dust with high quartz concentrations in a murine alveolar type II cell line (C10) [23,50]. In addition, *ATF3 *was significantly up-regulated by crystalline silica as early as 4 and up to 24 h (Figure 4B). In studies investigating gene expression induced by asbestos in human mesothelial and bronchial epithelial cells [31,32,42,51], as well as by cigarette smoke extract in 3T3 fibroblasts [52] and benzo(a)pyrine diol-epoxide (BPDE) in bronchial epithelial cells [42], *ATF3 *expression is significantly increased. Moreover, Shukla *et al*. [32], demonstrated that *ATF3 *expression was causally linked to the release of cytokines from human mesothelial cells exposed to asbestos fibers using siRNA approaches.

To our knowledge, up-regulation of *ATF3 *by crystalline silica particles has not been reported previously. It is possible, based on our earlier work [32] and results below, that *ATF3 *may be linked to secreted-mediator release from bronchial epithelial cells. For example, *IL6 *and *IL8*, which are linked to early inflammatory response, are up-regulated at the mRNA level and released from lung epithelial cells, alveolar macrophages, and in BALF of mice exposed to crystalline silica [21,45-48]. Our studies show that *IL6 *and *IL8 *mRNA levels are up-regulated significantly (p < 0.05) at 4, 8, and 24 h by cristobalite (Figure 4C, D). In summary, data in Figure 4 show that stress-response and pro-inflammatory genes are up-regulated early and over time, peaking at 4 h, in human bronchial epithelial cells by crystalline, but not amorphous silica particles. As both particle types are similar in size and chemical composition, non-toxic to, and taken up by BEAS 2B cells, their differential effects on gene expression may be related to differences in crystallinity.

### Cristobalite silica selectively induces pro-inflammatory and angiogenic cytokine and chemokine secretion from BEAS 2B cells

**Figure 5:**
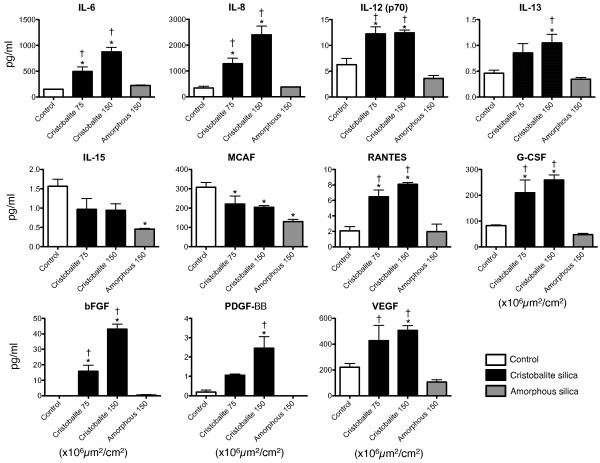
**Bio-Plex analysis of secreted cytokines and chemokines in medium of BEAS 2B cells exposed to silica particles 24 h**. Cell-free conditioned medium of BEAS 2B exposed to silica particles for 24 h was assayed for 27 cytokines and chemokines. Presented in this panel are the 11 which yielded significant differences from untreated controls in levels of secreted proteins: Interleukin-6 (IL-6), Interleukin-8 (IL-8), Interleukin-12 (p70) (IL-12 (p70)), Interleukin-13 (IL-13), Interleukin-15 (IL-15), Monocyte chemotactic protein-1 (MCP-1 or MCAF), Regulated upon activation, normal T-cell expressed and secreted (RANTES), Granulocyte-colony stimulating factor (G-CSF), Basic fibroblast growth factor (bFGF), Platelet derived growth factor-BB (PDGF-BB) and Vascular endothelial growth factor (VEGF). White bars (unexposed controls), black bars (cristobalite silica) and gray bars (amorphous silica), N = 3. * (P < 0.05) vs. unexposed controls, †P < 0.05 vs. amorphous silica.

Figure 5 shows the results of Bio-Plex assays. Levels of 9 cytokines/chemokines in medium were increased (p < 0.05) selectively by cristobalite and 2 were decreased by both silica types when compared to unexposed control cells. As for the remainder of the 27 cytokines and chemokines surveyed, 8 (IFNγ, IP-10, IL-1β, IL-7, IL-9, IL-10, MIP-1α and MIP-1β) yielded insignificant changes when compared to unexposed controls, and 8 (IL-1ra, IL-2, IL-4, IL-5, IL-17, Eotaxin, GM-CSF and TNFα) were below the lowest detection limit of the assay. Exposure of BEAS 2B cells to cristobalite at 75 and 150 × 10^6^μm^2^/cm^2 ^for 24 h caused dose-responsive and significant release of Interleukin-6 (IL-6), IL-8, IL-12 (p70), IL-13, regulated on activation normal T-cell expressed and secreted (RANTES), granulocyte colony-stimulating factor (G-CSF), basic fibroblast growth factor (bFGF/FGF-2), platelet-derived growth factor-BB (PDGF-BB), and vascular endothelial growth factor (VEGF) (Figure 5). Interestingly, amorphous silica, even at the high surface area concentration (150 × 10^6^μm^2^/cm^2^) did not induce increased secretion of any of these mediators. However, amorphous silica induced a significant decrease in the amount of interleukin-15 (IL-15) detected in medium, and both cristobalite and amorphous silica caused significant decreases in the amounts of monocyte chemotactic protein-1 (MCP-1; MCAF) secreted by BEAS 2B cells. Possible roles of IL-12 and IL-15 have been addressed in mouse inhalation models of silicosis [53]. However, it appears that innate immunity (as opposed to adaptive responses) plays a much stronger role in the development of silicosis [54], suggesting that IL-12 may have a less likely role in silica-induced disease.

It has been shown by others that crystalline silica particles induce expression and secretion of IL-6 and IL-8 [21,45-49], potent mediators promoting acute phase responses and inflammation in lung diseases [55-58], and thus are implicated in silicosis [59]. RANTES and MCP-1/MCAF are secreted mediators associated with recruitment of monocytes, lymphocytes, and granulocytes [60]. Increased levels of RANTES and MCP-1/MCAF expression also have been reported in alveolar type II cells exposed to crystalline silica [45], though increased expression of MCP-1 was seen early and had nearly diminished by 24 hours. Our studies show decreased levels of MCP-1/MCAF in medium of both crystalline and amorphous silica exposed BEAS 2B cells. The decreased levels of secreted MCP-1/MCAF may indicate a mechanism of lung defense or possibly an autocrine stimulation/negative feedback loop. In contrast, G-CSF is an extremely strong potentiator of neutrophilic response and recruitment (as reviewed in [61]), and is implicated in development of inflammation and pulmonary fibrosis [19].

In addition to modulators of inflammation, the proliferative and angiogenic factors, bFGF, VEGF and PDGF-BB were released from BEAS 2B cells exposed to cristobalite silica. PDGF-BB, a promoter of angiogenesis and cell proliferation, is implicated in silicosis and other pneumoconioses [62,63], and its expression may correlate with disease progression. Increased levels of serum VEGF are also observed in patients with idiopathic pulmonary fibrosis (IPF) [64]. In contrast, VEGF derived from myeloid cells is anti-fibrotic in a mouse model of pulmonary fibrosis [65]. Perhaps the cell of origin or location of VEGF secretion or the concentration of VEGF plays pivotal roles in the development and progression of pulmonary fibrosis.

Our data, consistent with other studies using crystalline silica in bronchial epithelial cells, shows that epithelial cells also have the ability to secrete bFGF in response to crystalline silica [66,67]. However, our study shows that crystalline silica as opposed to amorphous silica particles selectively induced bFGF secretion. This may indicate an autocrine or paracrine role of epithelial cell-derived bFGF in promoting fibrotic disease as it has been shown that bFGF promotes fibroblast proliferation in a transforming growth factor-β (TGF-β) model of epithelial-mesenchymal transition [68,69] and in the development and progression of interstitial pulmonary fibrosis [70]. Additionally, it has been shown that bFGF is associated with mast cells in human silicotic lungs [71].

Taken together, a number of pro-inflammatory, proliferative and angiogenic factors are released from lung epithelial cells, which are initial target cells in silica-induced disease. How these factors may interact with and regulate the airway environment is crucial in delineation of the role the epithelium plays in initiation and progression of silica-induced fibrosis. In addition, production and secretion of proliferative and angiogenic mediators may play a role in the potential carcinogenic effects of crystalline silica, by inducing excessive cell proliferation.

### Assessment of cell viability and gene expression induced by silica particles in primary human bronchial epithelial cells (NHBE)

**Figure 6:**
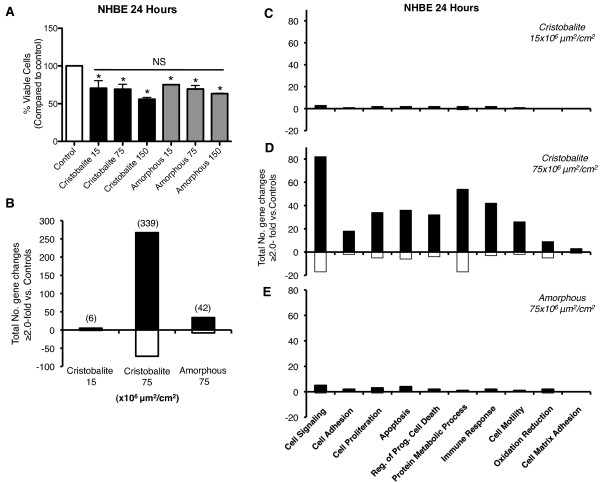
**Assessment of cell viability and microarray analysis of NHBE cells in response to silica particle exposure**. Effects of silica particles on NHBE cells after 24 h exposures. (**A**) Cell viability assessed by the Trypan blue exclusion assay (N = 3 in 2 independent experiments) *P < 0.05 compared to controls. NS = Not Significant for comparison of all exposure groups. (**B**) Total number of gene changes ≥ 2.0-fold vs. controls induced by silica particle exposure for 24 h. (**C-E**) Gene ontology analysis of NHBE exposed to (**B**) Cristobalite at 15 × 10^6^μm^2^/cm^2^, (**C**) 75 × 10^6^μm^2^/cm^2 ^and (**D**) amorphous silica at 75 × 10^6^μm^2^/cm^2^. Positive values represent number of genes up-regulated (Black bars) and negative values represent number of gene down-regulated (white bars) (N = 3).

NHBE cells were used to confirm results from experiments with BEAS 2B cells as these cells are primary as opposed to immortalized human bronchial epithelial cells and may be more responsive to particles. Figure 6 shows changes in cell viability and gene expression in primary NHBE cells by cristobalite and amorphous silica particles. When compared to BEAS 2B cells, NHBE cells were more sensitive to the toxicity of silica particles. After 24 h, both types of silica particles at 15, 75 and 150 × 10^6^μm^2^/cm^2 ^caused between 25-46% loss of viable cells (Figure 6A). Although particle exposures induced significant (p < 0.05) decreases in the percentage of viable cells compared to unexposed controls, no significant differences were observed between toxicity of cristobalite vs. amorphous silica at equal surface area concentrations. Based on these results, we chose to expose NHBE cells to cristobalite silica at the lowest (15) and a higher (75) surface area concentration and amorphous silica at 75 × 10^6^μm^2^/cm^2 ^in microarray analyses. Cristobalite at 15 × 10^6^μm^2^/cm^2 ^induced only 6 significant gene changes, though at the higher dose (75 × 10^6^μm^2^/cm^2^), 339 significant changes were detected. Interestingly, this is nearly the same number of significant changes (333) induced in BEAS 2B cells at the same exposure concentration (Figure 3A). In addition, amorphous silica at 75 × 10^6^μm^2^/cm^2^, (i.e. half the dose used in BEAS 2B cells) (Figure 3A), produced only 42 significant changes.

Gene ontology analysis of the gene expression alterations in NHBE cells exposed to silica particles was performed to compare the trends seen in BEAS 2B cells and to allow examination of similarities and differences between cells (Figure 6C-E, Additional file 3). The gene expression pattern of NHBE cells exposed to cristobalite or amorphous silica particles followed nearly the same patterns of expression with most gene changes categorized in the realms of cell signaling and proliferation, apoptosis, protein metabolic process and immune response.

Table 3 presents the top 10 genes up and down-regulated by cristobalite and amorphous silica particles in NHBE cells. *IL8*, at the top of this list, was up-regulated by cristobalite 4.37 and 28.47-fold at 15 and 75 × 10^6^μm^2^/cm^2 ^respectively. However, there was no increase in expression by amorphous silica particles. Interestingly, *TXNIP *was up-regulated by both cristobalite and amorphous silica by 9.74-fold and 31.14-fold respectively. This protein binds to NLRP3 and promotes inflammasome activation in response to oxidative stress [72]. Other genes of interest in this table include *BIRC3, BCL2A1 *and *TNFAIP3*, which are involved in regulation of apoptosis. When comparing the top 10 genes altered in expression by crystalline silica particles in BEAS 2B and NHBE cells, only one gene, Solute carrier family 38, member 4 (*SLC38A4*), a sodium-dependent amino acid symporter, is common to both cells (within the top 10). However, when all gene expression changes by cristobalite silica were compared in BEAS 2B and NHBE cells (Table 4), we found 60 genes commonly altered (57 up-regulated and 3 down-regulated). Of these 60 genes, 36 were altered in both cell types at the same surface area concentration (75 × 10^6^μm^2^/cm^2^) and 57 were commonly altered in NHBE cells at 75 × 10^6^μm^2^/cm^2 ^vs. BEAS 2B at 150 × 10^6^μm^2^/cm^2^. It should be noted that 60 genes may not appear to be a large portion of over 300 genes altered by cristobalite. However, the majority of these genes have strong connections with potential mechanisms of crystalline silica-induced disease, namely inflammation, fibrotic processes, as well as cell proliferation/anti-apoptosis (carcinogenesis).

**Table 3:** Top 10 genes affected by 24 h exposure to silica particles in NHBE

Gene Name (Abbreviation)	Fold Change^a^		
	Cristobalite		Amorphous
	15^b^	75^b^	75^b^
**Up-regulated ▲**			
Interleukin 8 (**IL8**)	4.37	28.47	NC
Baculoviral IAP repeat-containing 3 (**BIRC3**)	2.02	19.81	NC
BCL2-related protein A1 (**BCL2A1**)	NC	19.27	NC
Tumor necrosis factor, alpha-induced protein 3 (**TNFAIP3**)	2.33	17.11	NC
Interleukin 1 family, member 9 (**IL1F9**)	NC	14.14	NC
GTP binding protein over expressed in skeletal muscle (**GEM**)	NC	12.92	NC
CD83 molecule (**CD83**)	NC	11.47	NC
Transcribed locus, strongly similar to NP_006463.2 thioredoxin interacting protein	NC	11.09	27.95
Inhibin, beta A (**INHBA**)	NC	10.25	NC
Thioredoxin interacting protein (**TXNIP**)	NC	9.74	31.14
Heat shock 70 kDa protein 1A (**HSPA1A**)	2.01	7.56	2.57
Crystallin, mu (**CRYM**)	NC	4.97	2.35
G0/G1 switch 2 (**G0S2**)	NC	3.51	3.12
Transcribed locus (BF4328873)	NC	NC	2.73
Calcium binding tyrosine-(Y)-phosphorylation regulated (**CABYR**)	NC	2.51	2.55
Non-protein coding RNA 84 (**NCRNA00084**)	NC	NC	2.53
Sarcoglycan, gamma (35 kDa dystrophin-associated glycoprotein) (**SGCG**)	NC	NC	2.50
Cytoplasmic FMR1 interacting protein 2 (**CYFIP2**)	NC	NC	2.48

**Down-regulated ▼**			
Sulfatase (**SULF1**)	NC	3.59	NC
Peroxisome proliferator-activated receptor gamma, coactivator 1 alpha (**PPARGC1A**)	NC	3.52	NC
Solute carrier family 38, member 4 (**SLC38A4**)	NC	3.43	NC
Amyotrophic lateral sclerosis 2 (juvenile) chromosome region, candidate 8 (**ALS2CR8**)	NC	3.18	2.00
Growth arrest-specific 1 (**GAS1**)	NC	2.84	NC
Hexokinase 2 (**HK2**)	NC	2.69	NC
F-box protein 9 (**FBXO9**)	NC	2.68	NC
Hairy/enhancer-of-split related with YRPW motif 1 (**HEY1**)	NC	2.62	NC
CDNA FLJ37852 fis, clone BRSSN2014513	NC	2.61	NC
Growth hormone receptor (**GHR**)	NC	2.59	NC
Solute carrier family 16, member 7 (monocarboxylic acid transporter 2) (**SLC16A7**)	NC	2.50	NC
Transglutaminase 3 (E polypeptide, protein-glutamine-gamma-glutamyltransferase) (**TGM3**)	2.13	NC	NC
Follistatin (**FST**)	NC	NC	2.43
Homo sapiens clone FLB9440 PRO2550 mRNA, complete cds	NC	2.32	2.18
Ribonucleotide reductase M2 polypeptide (**RRM2**)	NC	NC	2.12
PPPDE peptidase domain containing 1 (**PPPDE1**)	NC	2.23	2.06

Top 10 genes in NHBE cells up and down-regulated by 24-hour exposure to cristobalite silica at 15 and 75 × 10^6 ^μm^2^/cm^2 ^and amorphous silica particles at 75 × 10^6 ^μm^2^/cm^2^.

**^a ^**Fold change (mRNA) expression (p < 0.05) cut-off ≥ 2.0-fold compared to controls (n = 3).

**^b ^**Silica particle concentrations: (× 10^6^μm^2^/cm^2^)

NC = No Change

**Table 4:** Genes commonly affected by 24 h cristobalite exposure in NHBE and BEAS 2B cells

Gene Name (Abbreviation)	Fold Change^a^		
Cell Type (Cristobalite)^b^	NHBE (75)	BEAS 2B (75)	BEAS 2B (150)
**Up-regulated ▲**			
Interleukin 8 (**IL8**)	28.47	2.12	2.65
BCL2-related protein A1 (**BCL2A1**)	19.27	NC	3.08
Tumor necrosis factor, alpha-induced protein 3 (**TNFAIP3**)	17.11	2.23	2.95
GTP binding protein overexpressed in skeletal muscle (**GEM**)	12.92	NC	2.13
Inhibin, Beta A (**INHBA**)	10.25	2.24	3.62
Interleukin 24 (**IL24**)	7.62	3.88	8.54
Heat shock 70 kDa protein 1A (**HSPA1A**)	7.56	NC	2.34
Chemokine (C-X-C motif) ligand 3 (**CXCL3**)	7.42	NC	2.42
TNF receptor-associated factor 1 (**TRAF1**)	7.30	2.86	4.22
Radical S-adenosyl methionine domain containing 2 (**RSAD2**)	6.92	5.45	23.25
Early growth response 1 (**EGR1**)	6.72	2.65	4.63
Coiled-coil domain containing 85B (**CCDC85B**)	6.46	3.44	6.11
FOS-like antigen 1 (**FOSL1**)	5.80	2.47	3.18
Early growth response 3 (**EGR3**)	5.31	NC	2.65
Jun oncogene (**JUN**)	4.99	NC	2.00
Matrix metallopeptidase 1, interstitial collagenase (**MMP1**)	4.99	16.12	44.68
2'-5'-oligoadenylate synthetase-like (**OASL**)	4.20	NC	18.85
cAMP responsive element binding protein 5 (**CREB5**)	3.87	3.25	4.10
Transcribed locus, strongly similar to NP_000337.1 transcription factor SOX9 (**SOX9**)	3.84	NC	2.12
Interferon induced with helicase C domain 1(**IFIH1**)	3.79	NC	3.86
Heat shock 70 kDa protein 6 (**HSP70B'**)	3.70	4.08	14.39
Kynureninase (L-kynurenine hydrolase) (**KYNU**)	3.66	NC	2.19
Prostaglandin-endoperoxide synthase 2 (**PTGS2**)	3.56	NC	2.54
Superoxide dismutase 2, mitochondrial (**SOD2**)	3.53	NC	2.86
Interleukin 1 receptor-like 1 (**IL1RL1**)	3.52	2.89	5.36
Interferon-induced protein with tetratricopeptide repeats 1 (**IFIT1**)	3.45	6.01	16.26
Interferon-induced protein 44 (**IFI44**)	3.36	7.51	19.90
Nuclear receptor subfamily 1, group D, member 1 (**NR1D1**)	3.24	3.01	4.72
Interferon stimulated exonuclease gene 20 kDa (**ISG20**)	3.21	NC	2.48
Plasminogen activator, urokinase receptor (**PLAUR**)	3.14	NC	2.35
Transmembrane protein 40 (**TMEM40**)	3.06	NC	4.15
Distal-less homeobox 2 (**DLX2**)	2.99	2.34	3.78
Niacin receptor 2 (**NIACR2 **OR **GPR109B**)	2.87	3.14	NC
Human 28S ribosomal RNA gene, complete cds. (**RNA28S1**)	2.86	NC	2.21
Histone deacetylase 9 (**HDAC9**)	2.84	3.24	NC
2'-5'-oligoadenylate synthetase 1, 40/46 kDa (**OAS1**)	2.83	NC	20.53
Nuclear receptor subfamily 4, group A, member 3 (**NR4A3**)	2.79	2.58	3.49
Oxidative stress induced growth inhibitor 1 (**OSGIN1**)	2.79	NC	2.48
Smoothelin (**SMTN**)	2.74	2.38	2.72
Heme oxygenase (decycling) 1 (**HMOX1**)	2.69	NC	2.57
Basic helix-loop-helix family, member e41 (**BHLHE41**)	2.64	NC	2.34
ADAM metallopeptidase domain 8 (**ADAM8**)	2.62	NC	2.08
Crystallin, alpha B (**CRYAB**)	2.58	2.26	2.67
Colony stimulating factor 2 (granulocyte-macrophage) (**CSF2**)	2.53	4.20	7.37
Calcium binding tyrosine-(Y)-phosphorylation regulated (**CABYR**)	2.51	NC	2.19
Chromosome 1 open reading frame 38 (**C1orf38**)	2.44	NC	3.04
2'-5'-oligoadenylate synthetase 3, 100 kDa (**OAS3**)	2.29	2.41	3.84
Sprouty homolog 4 (Drosophila) (**SPRY4**)	2.26	2.98	4.64
Dual specificity phosphatase 5 (**DUSP5**)	2.23	2.02	2.61
Ring finger protein 39 (**RNF39**)	2.22	2.47	3.65
Leukemia inhibitor factor (Cholinergic differentiation factor) (**LIF**)	2.21	2.96	4.73
Sphingosine kinase 1 (**SPHK1**)	2.11	3.20	4.88
Interleukin 6 (Interferon, beta 2) (**IL6**)	2.10	2.12	2.59
Interleukin 1, alpha (**IL1A**)	2.09	3.97	6.17
FBJ murine osteosarcoma viral oncogene homolog B (**FOSB**)	2.07	3.65	6.37
Serpin peptidase inhibitor, clade B (ovalbumin), member 2 (**PAI2**)	2.04	2.16	4.33
DEAD (Asp-Glu-Ala-Asp) box polypeptide 60 (**DDX60**)	2.02	2.26	5.01

**Down-regulated ***▼*			
Sulfatase 1 (**SULF1**)	3.59	2.01	2.10
Peroxisome proliferator-activated receptor gamma, coactivator 1 alpha (**PPARGC1A**)	3.52	2.85	3.75
Chromosome 5 open reading frame 13 (**C5orf13**)	2.05	NC	2.21

Sixty genes (57 up-regulated and 3 down-regulated) found commonly altered by exposure to cristobalite silica in NHBE cells and BEAS 2B cells from microarray analysis. Genes are listed in descending order of fold-change compared to controls seen in NHBE cells.

**^a ^**Fold change (mRNA) expression (p < 0.05) cut-off ≥ 2.0-fold compared to controls (n = 3)

**^b ^**Cristobalite silica particle concentrations: (× 10^6^μm^2^/cm^2^)

NC = No Change

Within this list of commonly affected genes there are a number of transcription factors, cytokines/chemokines and receptors, oxidative stress-related, and genes related to the regulation of apoptosis, proliferation, cell signaling and ECM breakdown. The transcription factors, *FOSL1 (fra-1), FOSB *and*, JUN*, members of the AP-1 complex family, as well as *CREB5*, which can also interact with *jun *family members, were up-regulated by cristobalite in both cell lines. The AP-1 transcription factor complex appears to play a major role in cellular response to crystalline silica [50]. In addition, the transcriptional regulators *EGR1 *and *EGR3 *were induced by cristobalite silica.

A number of pro-inflammatory mediators were also increased at the mRNA level by cristobalite in NHBE and BEAS 2B cells including, *IL1A, IL6, IL8, IL24*, *CXCL3*, (MIP-2β), *CSF2 *(GM-CSF) and the *IL1RL1*. Some of these genes (*IL1A, IL1RL1*) and their proteins are involved in regulation and activity of the NALP3 inflammasome, a signaling complex that responds to "danger signals" and promotes an inflammatory response after addition of silica to human THP-1 macrophages [73]. In line with the hypothesis that oxidative stress mediates inflammasome activity by silica, oxidative-stress response-genes, *PTGS2 *(COX2), *SOD2 *(MnSOD), *HMOX1 and OSGIN1 *were also up-regulated by cristobalite in NHBE and BEAS 2B cells. These findings also reinforce the observations that inflammation and oxidative stress are two of the driving forces in silica-induced disease mechanisms [74]. Inflammation and oxidative stress create an imbalanced cellular environment, with cellular injury, and death or cell proliferation. These mechanisms may contribute to airway remodeling and indirect mechanisms of carcinogenicity, promoting a favorable microenvironment for both fibrosis and tumor formation.

A number of other genes were also altered in expression by cristobalite in both cell lines, including genes involved in ECM breakdown (*MMP1 *and *PLAUR*), genes involved in cell signaling regulation (*HDAC9*), and regulators of MAPK signaling (*SPRY4 *and *DUSP5*). Other genes are involved in anti-viral activity and interferon-response including, *RSAD2, OAS1, OAS3, OASL, IFIH1, IFIT2, IFI44 and ISG20*. Of those genes, *IFIT1 *and *IFI44 *were up-regulated in both cell types by amorphous silica particles as well.

## Conclusions

Particulates, especially in the realm of nanotoxicology, are rapidly becoming a question of concern regarding health effects in occupational setting as well as in the environment. Our studies and a recent report in the literature [41] demonstrate that gene expression profiling in human lung epithelial cells *in vitro *can be used to determine the relative pathogenicity of potentially harmful particulates (i.e. crystalline silica and airborne particulate matter, respectively) as opposed to viability studies and metabolic testing. Changes in expression of genes related to inflammation, oxidative stress, and proliferation as well as the secretion of proinflammatory, angiogenic and proliferative cytokines and chemokines re-emphasize the complex reactions of the lung epithelium to crystalline silica particles and their relation to development of fibrosis [12].

Our studies compared two particles of the same chemical make up (SiO_2_), but of different habit (i.e. crystalline vs. amorphous). The responses observed suggest that the crystallinity of cristobalite may dictate early molecular responses as well as dissolution of particles over time in the lung. Moreover, we show that the magnitude and patterns of differential gene expression by crystalline silica are similar in both BEAS 2B and NHBE cells, suggesting that immortalized and established epithelial cell lines can be used as *in vitro *models to predict the pathogenicity of potentially harmful respirable particulates as opposed to expensive and time-consuming isolation of human primary cells and animal inhalation studies.

## Methods

### Human Bronchial Epithelial Cell Cultures

Non-tumorigenic human bronchial epithelial cells (Ad12-SV40 immortalized) BEAS 2B (ATCC, Manassas, VA) were grown and maintained in DMEM/F12 50:50 media containing 10% Fetal Bovine Serum (FBS) (CellGro^® ^Mediatech inc, Manassas, VA), with penicillin (50 units/ml), streptomycin (100 μg/ml) (Invitrogen, Carlsbad, CA), hydrocortisone (100 μg/ml), insulin (2.5 μg/ml), transferrin (2.5 μg/ml) and selenium (2.5 μg/ml) (Sigma, St. Louis, MO). BEAS 2B cells culture flasks and plates (BD, Franklin Lakes, NJ) were pre-coated with a mixture of fibronectin (Sigma, St. Louis, MO) (0.01 mg/ml), bovine collagen type I (0.03 mg/ml) (Invitrogen, Carlsbad, CA) and bovine serum albumin (0.01 mg/ml) (Sigma, St. Louis, MO), dissolved in DMEM/F12 media for 24 hours at 37°C prior to culturing cells. Prior to exposures, medium was aspirated and replaced with a reduction medium containing 0.5% FBS. Primary normal human bronchial epithelial cells (NHBE-17917) were purchased from Lonza, Clonetics ^®^. NHBE cells were cultured and maintained in BEGM^® ^and with Reagentpack™subculture reagents (Trypsin/EDTA, Trypsin Neutralizing Solution and Hepes-buffered saline solution), all purchased from Lonza, Clonetics ^®^. (Switzerland) were used for subculture following manufacturers protocol.

### Synthesis, Characterization, and Determination of Surface Areas of Silica Particles

The amorphous silica microparticles were prepared according to the procedure of De et al [75] in the Department of Chemistry, University of Vermont, Burlington, VT, USA. Briefly, tetraethylorthosilicate (32.0 g, 0.143 mol) was combined with water (11.0 g, 0.611 mol) and stirred rapidly (600 rpm) to form an emulsion. To this emulsion was rapidly added glacial acetic acid (36.8 g, 0.643 mol), and stirring was continued for 60 s, at which point stirring was terminated. The mixture was then allowed to sit quiescently for 45 min. Afterward the particles were filtered and washed extensively with water (5 mL × 4) and ethanol (5 mL × 3). After air-drying for several hours, the particles were dried overnight under vacuum, then particles were calcined at 550°C for 12 hours under air. Finally, the particles were ground with a mortar and pestle to yield a freely-flowing, white powder (4.8 g, 56% yield). Samples were mounted for SEM by dusting onto carbon tape applied to aluminum sample stubs. They were then sputter coated with Pd/Au for 3.5 minutes in a Polaron sputter coater (Model 5100). Specimens were then examined with a JSM 6060 scanning electron microscope (JEOL USA, Inc., Peabody, MA) operating at an accelerating voltage of 25 kV and a working distance of 10 mm. Size distribution was determined using SEM images by measuring the diameter of 300-400 silica particles (5 fields of view) using Metamorph offline software version 7.7.3 64 bit (Molecular Devices, Sunnyvale, CA). Cristobalite silica was examined by powder X-ray diffraction methods using a Rigaku MiniFlexII X-ray diffractometer. Rietveld analysis, as implemented in the Rigaku WPPF program, was undertaken with eight-second fixed time scans at 0.02° steps from 10° to 100° 2θ. For the scan, R*wp *= 10.94%, modelled with α-quartz and cristobalite. The quantitative analysis by Rietveld analysis yielded 95.5(± 0.03)% cristobalite, and 4.54(0.08)% quartz. Surface area of particles was determined by BET nitrogen adsorption analysis. Nitrogen adsorption and desorption isotherms were obtained on a Micromeritics TriStar instrument after samples were degassed overnight under vacuum. Surface areas were measured using the BET method, and pore size distributions were calculated from a modified KJS method using the adsorption branch [76-78].

### Particle Exposure to Cells

Prior to exposure, particles were weighed in scintillation vials and placed under UV light overnight to be sterilized. Particles were then submerged in Hanks' Balanced Salt Solution (HBSS) (CellGro^® ^Mediatech inc, Manassas, VA) at (1.0-2.0 mg/ml). Particle suspensions were sonicated in a waterbath sonicator for 15 minutes followed by being trichurated 5 times through a 22-gauge needle. Suspensions were then administered to cell culture plates and briefly shaken to assure dispersion of particles before they settle.

### Assessment of Cell Viability

After 24 h, cells were collected by detatching with 0.25% Trypsin (Invitrogen, Carlsbad, CA) solution in HBSS, and a final volume of 1.5 ml (0.5 ml trypsin solution + 1.0 ml 10% FBS medium was collected on ice. This solution was diluted 1:5 in a solution of 0.4% trypan blue (MP Biomedicals, Solon, OH), which is a dye retained by dead cells and excluded by viable cells and HBSS. After 5 min, unstained viable cells were counted using a hemocytometer to determine the total number of viable cells per dish and particle exposed groups were compared unexposed controls as described previously [79] For each group (particle type/concentration) n = 3 and experiments were performed in triplicate (BEAS 2B) and duplicate (NHBE).

### Scanning Electron Microscopy

For imaging of cristobalite silica and amorphous silica particles, 0.0029 or 0.0026 g was diluted to a final concentration of 1.45 and 1.3 μg/ml (4.0 ml total volume), respectively, in a solution of 6% ethanol and ddH_2_O by serial dilution. Silica particle dilutions were filtered through a 0.4 μm Nucleopore Track-Etch membrane (Fisher Scientific, Pittsburgh, PA) followed by a rinse with 1.0 ml 100% ethanol and drying overnight. Half of the dried filter was adhered to a standard aluminum specimen stub with graphite point (Electron Microscope Sciences, Hatfield, PA) followed by sputter coating with gold and palladium using a Polaron sputter coater (Model 5100; Quorum Technologies, East Sussex, UK) Specimens were imaged and EDS analysis was performed using an INCA 350 × Max detector and INCA Energy System software (Oxford Instruments, Concord, MA) by "point-analysis". BEAS 2B cells were grown on Thermonox plastic cover slips (Nalge Nunc International, Naperville, IL). Prior to seeding cells, cover slips were coated with fibronectin (0.01 mg/ml), bovine collagen type I (0.03 mg/ml) and bovine serum albumin (0.01 mg/ml) dissolved in DMEM/F12 medium for 24 h at 37°C. Cells were grown to near confluency and then put in reduction medium (0.5% FBS) 24 h prior to incubation with particles. Cells were exposed to silica particles for 2 and 24 h before fixation. Cells were fixed with 2% glutaraldehyde in 0.1 M phosphate buffered saline (PBS) for 1 h at 4°C. Samples then rinsed for 5 min in PBS (3×) and then (1×) 5 minutes in 0.05 M cacodylate buffer (pH 7.2). Samples then post-fixed with 1% osmium tetroxide in 0.05 M cacodylate buffer (pH7.2) for 1 h at room temperature, then rinsed (3×) for 5 min in 0.05 M cacodylate buffer. Samples immersed in 1% tannic acid in 0.05 M cacodylate buffer for 1 h at room temperature and in the dark (covered with foil), rinsed 1× for 5 min in cacodylate buffer and 3× for 5 min in distilled water. Samples incubated with 0.5% uranyl acetate in distilled water for 1 hr at room temperature in the dark (covered with foil), rinsed 3× for 5 min in distilled water, and stored in 0.05 M cacodylate buffer at 4°C. Cover slips were put in cylindrical holder and dehydrated in ethanol series: using 10 min changes in 35, 50, 70, 85% up to 95% ethanol, then (2×) 20 min changes in 95% ethanol, followed by three 15 min changes in anhydrous 100% ethanol. Samples were then critical-point dried and fixed to aluminium specimen mounts with carbon paint and dried overnight in a desiccator. Samples were then sputter-coated with gold and palladium in a Polaron sputter coater (Model 5100) and imaged on JSM-6060 scanning electron microscope (JEOL USA, Inc. Peabody, MA).

### RNA Isolation and Microarray Profiling

Total RNA was prepared using an RNeasy^® ^Plus Mini Kit according to the manufacturers' protocol (Qiagen, Valencia, CA), as published previously [80]. All procedures were performed by the Microarray analysis core facility of the Vermont Genetics Network (VGN) using a standard Affymetrix protocol as described previously [80,81]. GeneChip^® ^Human Genome U133A 2.0 arrays (Affymetrix, Santa Clara, CA) targeting 18,400 human transcripts were scanned twice (Hewlett-Packard GeneArray Scanner), the images overlaid, and the average intensities of each probe cell compiled. Microarray data were analyzed using GeneSifter software (VizX Labs, Seattle, WA). This program used a t-test for pair- wise comparison and a Benjamini-Hochberg test for false discovery rate (FDR 5%) to adjust for multiple comparisons. A 2-fold cutoff limit was used for analysis (only genes up or down-regulated ≥ 2.0-fold vs. unexposed controls)

### Quantitative Real-time Polymerase Chain Reaction (qRT-PCR)

Total RNA (1 μg) was reverse-transcribed with random primers using the AMV Reverse Transcriptase kit (Pro-mega, Madison, WI) according to the recommendations of the manufacturer, as described previously [80]. All Taqman^® ^qRT-PCR was performed using Assays-On-Demand™ primer and probe sets used for Applied Biosystems (Foster City, CA) and performed as described previously [31].

### Bio-Plex Analysis of Cytokines and Chemokines in Medium of BEAS 2B Cells

To quantify cytokine, chemokine, and growth factor levels in conditioned medium of BEAS 2B cells exposed to silica particles, a multiplex suspension protein array was performed using the Bio-Plex^® ^protein array system and a human cytokine 27-plex panel (Bio-Rad, Hercules, CA), as previously described [80]. A sample of 0.05% FBS medium alone was also run to account for background levels of secreted proteins. Data are expressed as pg of cytokine/mL of conditioned medium (mean ± SEM), with N = 3 for each group.

### Statistical Analyses

Statistical analysis of results from cell viability and Bio-Plex analysis were evaluated by analysis of variance (ANOVA) using the Student Neuman-Keul's procedure for adjustment of multiple pair-wise comparisons between treatment groups. Significant differences in gene expression values determined by qRT-PCR were evaluated using a Student's t-test. Differences with p values < 0.05 were considered statistically significant.

## Competing interests

The authors declare that they have no competing interests.

## Authors' contributions

The study design was constructed by TNP, BTM, NLR and EFW. Cell culture, RNA isolation, medium collection, cDNA preparation was performed by TNP and PMP. TNP and AS performed the majority of data analysis. JLS and CCL synthesized amorphous silica particles and determined surface area properties of particles. SLM performed SEM. CS performed Bio-Plex analysis cell supernatants. TNP and BTM drafted the manuscript. All authors have read and approved the final manuscript.

## Supplementary Material

Additional file 1 **SEM Imaging and EDS Analysis of Silica Particles**. SEM images of filtered (**A**) cristobalite silica and (**B**) amorphous silica particles with respectful electron dispersive spectroscopy (EDS) spectra showing their identical chemical composition. Images are at a magnification of 1500× and scale bars equal to 10 μm. Black arrows indicate the points analyzed by EDS "point-analysis", spectra represent points indicated. Silicon (Si) and oxygen (O) peaks of each particle type indicate both are pure silica particles. Gold (Au) and palladium (Pd) peaks are present due to the gold and palladium sputter-coating, carbon (C) is present because of graphite paint used to mount particle filters. Click here for file

Additional File 2 **Silica Particle Size Distrbution**. Histograms represent size-distribution of cristobalite silica particles (**A**) and amorphous silica particles (**B**). Particles were filtered and imaged by SEM at 1000× magnification. The diameter of each particle was recorded using Metamorph^®^, and 300-400 particles (5 fields/stub) were measured for each type of silica particle. Click here for file

Additional file 3 **Gene Ontology of BEAS 2B and NHBE Exposed to Silica Particles for 24 h (%Total/Category)**. Pie charts (**A-F**) represent gene ontology analysis of alterations in gene expression of BEAS 2B cells (**A-C**) exposed to (**A**) cristobalite 75 × 10^6^μm^2^/cm^2^, (**B**) cristobalite 150 × 10^6^μm^2^/cm^2 ^and (**C**) amorphous silica 150 × 10^6^μm^2^/cm^2^. Gene ontology of NHBE cells (**D-F**) exposed to (**D**) cristobalite 15 × 10^6^μm^2^/cm^2^, (**E**) cristobalite 75 × 10^6^μm^2^/cm^2 ^and amorphous silica 75 × 10^6^μm^2^/cm^2^. Ten categories of interest were analyzed and charts represent the percent (%) of total genes altered in each category for each exposure group. Click here for file
