# Supplementary figures and images for: Differences in gene expression and cytokine production by crystalline vs. amorphous silica in human lung epithelial cells
Source: Part Fibre Toxicol. 2012 Feb 2;9:6. doi: 10.1186/1743-8977-9-6 (PMC3337246; doi:10.1186/1743-8977-9-6)

**A**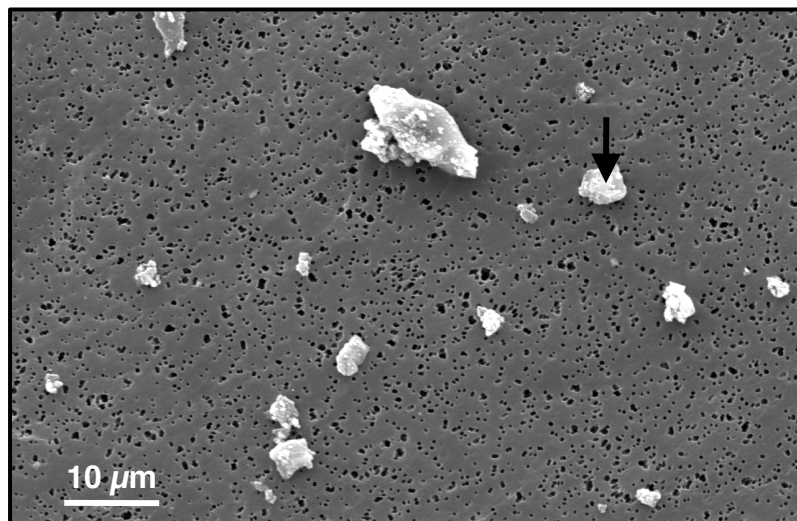

Spectrum 1

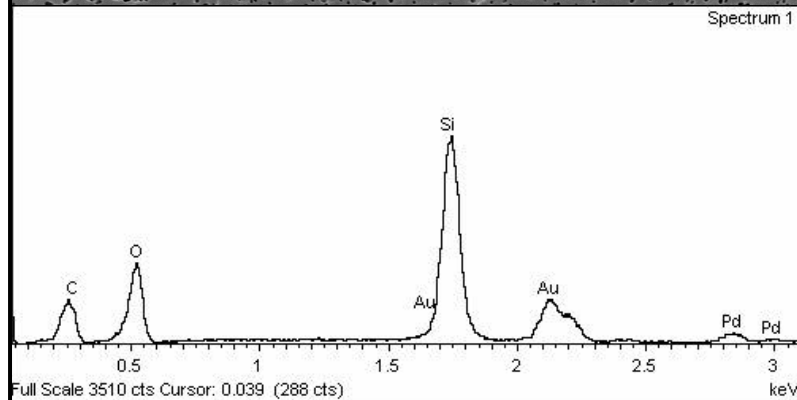**B**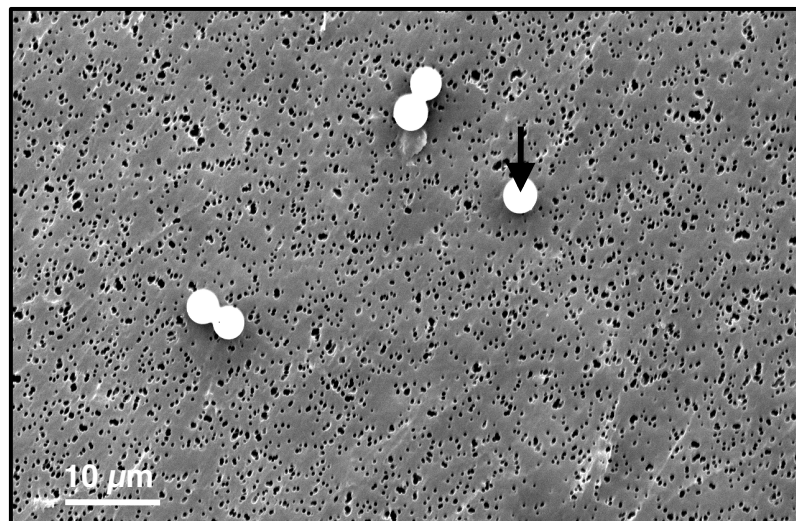

Spectrum 1

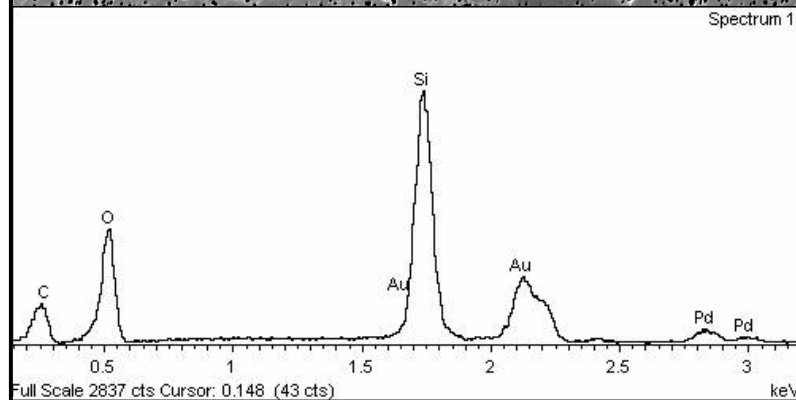

Supplement: Additional file 1 — SEM Imaging and EDS Analysis of Silica Particles. SEM images of filtered (A) cristobalite silica and (B) amorphous silica particles with respectful electron dispersive spectroscopy (EDS) spectra showing their identical chemical composition. Images are at a magnification of 1500× and scale bars equal to 10 μm. Black arrows indicate the points analyzed by EDS "point-analysis", spectra represent points indicated. Silicon (Si) and oxygen (O) peaks of each particle type indicate both are pure silica particles. Gold (Au) and palladium (Pd) peaks are present due to the gold and palladium sputter-coating, carbon (C) is present because of graphite paint used to mount particle filters. [file 1743-8977-9-6-S1.PDF]

**A****Cristobalite Silica**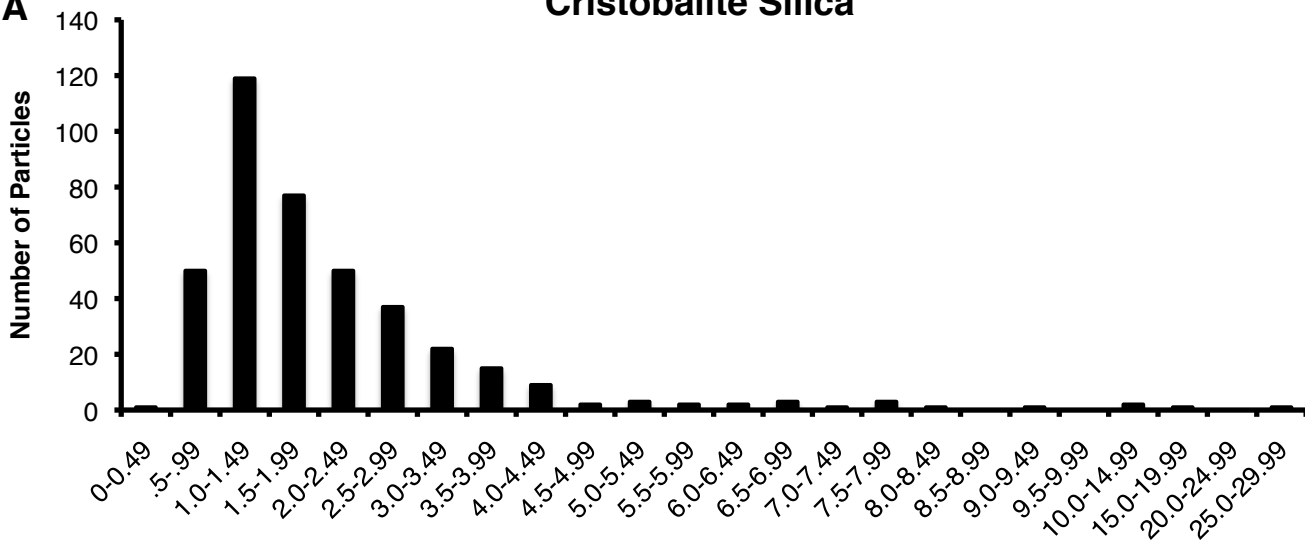**B****Amorphous Silica**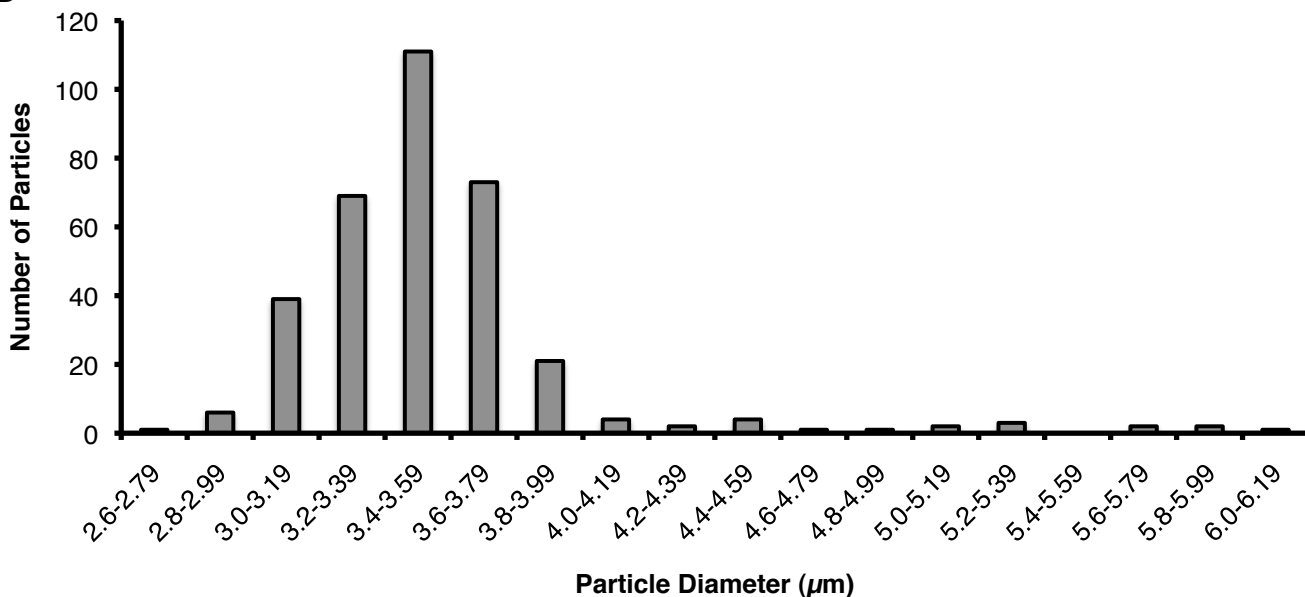

Supplement: Additional File 2 — Silica Particle Size Distrbution. Histograms represent size-distribution of cristobalite silica particles (A) and amorphous silica particles (B). Particles were filtered and imaged by SEM at 1000× magnification. The diameter of each particle was recorded using Metamorph®, and 300-400 particles (5 fields/stub) were measured for each type of silica particle. [file 1743-8977-9-6-S2.PDF]
