# Supplementary material for: Differences in gene expression and cytokine production by crystalline vs. amorphous silica in human lung epithelial cells
Source: Part Fibre Toxicol. 2012 Feb 2;9:6. doi: 10.1186/1743-8977-9-6 (PMC3337246; doi:10.1186/1743-8977-9-6)
Supplement: Additional file 3 — Gene Ontology of BEAS 2B and NHBE Exposed to Silica Particles for 24 h (%Total/Category). Pie charts (A-F) represent gene ontology analysis of alterations in gene expression of BEAS 2B cells (A-C) exposed to (A) cristobalite 75 × 106μm2/cm2, (B) cristobalite 150 × 106μm2/cm2 and (C) amorphous silica 150 × 106μm2/cm2. Gene ontology of NHBE cells (D-F) exposed to (D) cristobalite 15 × 106μm2/cm2, (E) cristobalite 75 × 106μm2/cm2 and amorphous silica 75 × 106μm2/cm2. Ten categories of interest were analyzed and charts represent the percent (%) of total genes altered in each category for each exposure group. [file 1743-8977-9-6-S3.PDF]

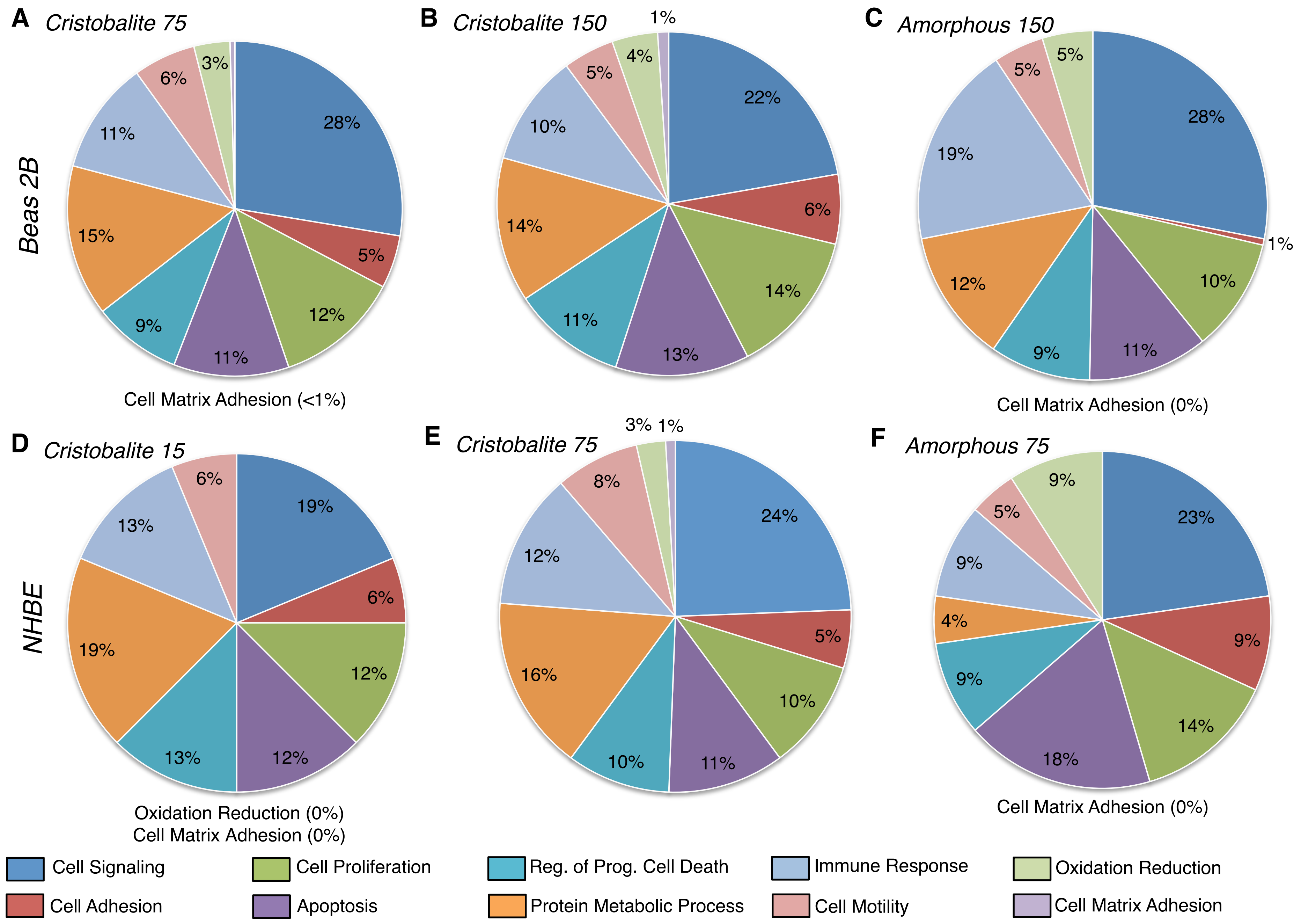
A
Cristobalite 75
28%
5%
12%
11%
9%
15%
11%
6%
3%
Cell Matrix Adhesion (<1%)
B
Cristobalite 150
1%
22%
6%
14%
13%
11%
14%
10%
5%
4%
C
Amorphous 150
28%
1%
10%
11%
9%
12%
19%
5%
5%
Cell Matrix Adhesion (0%)
Beas 2B
D
Cristobalite 15
19%
6%
12%
12%
13%
19%
13%
6%
Oxidation Reduction (0%)
Cell Matrix Adhesion (0%)
E
Cristobalite 75
3% 1%
24%
5%
10%
11%
10%
16%
12%
8%
F
Amorphous 75
23%
9%
14%
18%
9%
4%
9%
5%
9%
Cell Matrix Adhesion (0%)
NHBE
Cell Signaling
Cell Adhesion
Cell Proliferation
Apoptosis
Reg. of Prog. Cell Death
Protein Metabolic Process
Immune Response
Cell Motility
Oxidation Reduction
Cell Matrix Adhesion
